# Microencapsulation of the Biocide Benzisothiazolinone (BIT) by Inclusion in Methyl-β-cyclodextrin and Screening of Its Antibacterial and Ecotoxicity Properties

**DOI:** 10.3390/toxics12090674

**Published:** 2024-09-16

**Authors:** Vânia F. M. Silva, Aurora Silva, Ermelinda M. P. J. Garrido, Fernanda Borges, Alexandra Gaspar, Jorge M. P. J. Garrido

**Affiliations:** 1 CIETI, ISEP, Polytechnic of Porto, 4249-015 Porto, Portugal; vfmsi@isep.ipp.pt (V.F.M.S.); emg@isep.ipp.pt (E.M.P.J.G.); 2 Nutrition and Bromatology Group, Department of Analytical and Food Chemistry, Faculty of Food Science and Technology, University of Vigo, Ourense Campus, 32004 Ourense, Spain; mass@isep.ipp.pt; 3 REQUIMTE/LAQV, ISEP, Polytechnic of Porto, 4249-015 Porto, Portugal; 4 CIQUP-IMS, ISEP, Polytechnic of Porto, 4249-015 Porto, Portugal; 5 CIQUP-IMS, Department of Chemistry and Biochemistry, Faculty of Sciences, University of Porto, 4169-007 Porto, Portugal; fborges@fc.up.pt

**Keywords:** biocidal active substances, isothiazolinone, host–guest complex, encapsulation efficiency, biological activity

## Abstract

The excessive use of biocides has considerable environmental and economic impacts; this is why new technologies have been sought to decrease the concentration levels applied in an effort to reduce the use of these substances. Microencapsulation using cyclodextrins has been widely used in the food and pharmaceutical industries as a way of reducing the concentrations of the active substance necessary to achieve a biological effect and/or eliminate its irritating or toxicological effects. In this study, the inclusion complexation behavior and binding ability of benzothiazolinone (BIT) with different β-cyclodextrins (β-CD, HP-β-CD, and Me-β-CD) was investigated. The intermolecular interactions were examined through UV and FTIR spectroscopy, DSC, 1D ^1^H NMR, and 2D ROESY. The highest stability constant was observed for the BIT/Me-β-CD inclusion complex (299.5 ± 2.9 M^−1^). Antibacterial activity was investigated against *Staphylococcus aureus* and *Escherichia coli,* and the results revealed that the BIT/Me-β-CD inclusion complex displays a higher antibacterial activity than BIT. The acute toxicity of the biocide and inclusion complex was also examined using the photobacterium *Aliivibrio fischeri*. Although BIT exhibited higher toxicity than the inclusion complex, further investigation is needed due to the quorum quenching effect of β-CDs. The data found suggest that BIT microencapsulation can increase its aqueous solubility and can be used as an effective tool to improve its chemical, biological, and ecotoxicological properties.

## 1. Introduction

Biocidal products have been widely used for a long time and remain among the most effective solutions for the control of unwanted organisms that are harmful to human or animal health or that cause damage to natural or manufactured materials [1]. Biocidal products, due to their inherent properties, can pose harm to humans, animals, and the environment. As a result, the EU has established stringent rules and procedures to minimize these risks [2]. The excessive use, misuse, and increased doses to overcome resistance to biocides have significant environmental, economic, and public health impacts [3].

**Figure 1:**
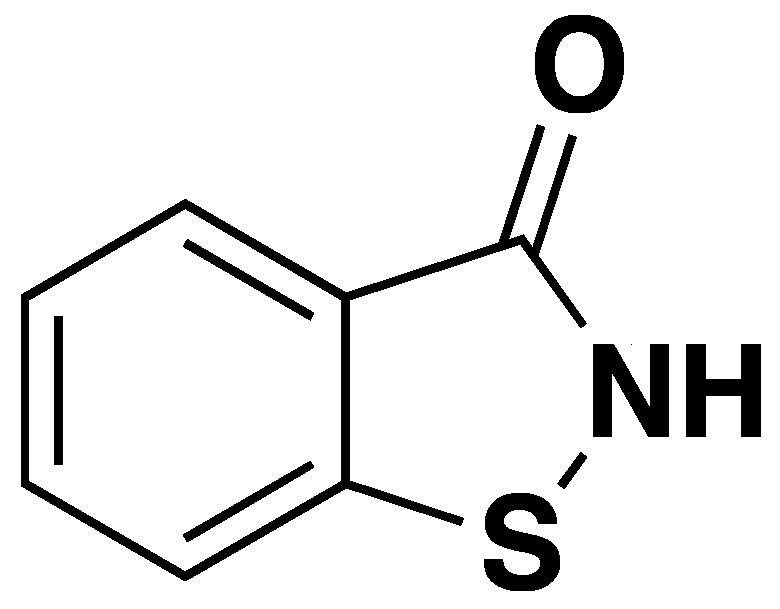
Chemical structure of benzisothiazolinone (BIT).

Isothiazolinones are a group of well-known biocides used as additives in a wide range of industrial products [4]. Among them, benzisothiazolinone (BIT) (Figure 1) is an antimicrobial agent used to preserve water-borne paints, varnishes, adhesives, sealants, and other products [5]. Although banned in the EU for cosmetic purposes, BIT is still used in household cleaning products and certain tanned leather and plastics [4,5,6]. The increased utilization of BIT and isothiazolinones in recent years, along with human exposure to BIT through dermal absorption, has raised multiple concerns about their potential to induce sensitization and allergic contact dermatitis [7]. Recent data have shown a significant increase in the prevalence of contact allergy to BIT, probably due to increased use in household products [5]. Additionally, it has been noted that BIT is very toxic to aquatic organisms and can induce blood–brain barrier dysfunction, which might contribute to the risk of cardiovascular or neurological diseases [8,9].

The growing focus on isothiazolinones and BIT, aiming to mitigate their adverse effects on health and the environment, has prompted more stringent regulations, reducing their allowable concentrations in several products. An approach that can be used to boost biocidal efficacy and reduce the inherent toxicity of BIT and other isothiazolinones is to incorporate them into formulations of one or more constituents [10,11]. In fact, the literature suggests that combining cyclodextrins with biocides can result in synergism between maintaining biocidal activity and minimizing environmental drawbacks [11,12,13]. The greater efficacy and potency caused by CD-increased drug solubility may reduce toxicity by making the biocide effective at lower doses [14].

Cyclodextrins (CDs) are cyclic oligosaccharides made up of multiple α-(1,4)-linked D-glucopyranose units. They have a unique three-dimensional structure, with a hydrophilic outer surface and a lipophilic central cavity. This structure enables CDs to partially or fully enclose appropriately sized hydrophobic molecules, forming inclusion complexes [15,16,17]. The capability to form inclusion complexes with a diverse range of guest molecules can positively impact many of the physicochemical properties of the entrapped molecules, thus offering numerous potential benefits such as improved aqueous solubility, stability, and bioavailability [15]. Moreover, CD complexation can also be used to enhance bioactive molecules’ shelf-life, reduce the concentrations of the agent required to achieve a biological effect, reduce/eliminate irritant or toxicological side-effects, and enhance the antimicrobial effectiveness of biocide agents [15,17,18].

The main aim of this study was to investigate the formation of water-soluble inclusion complexes of BIT with both native and modified β-cyclodextrins. Furthermore, this study also analyzes how complexation with CDs affects the solubility of BIT in water, and seeks to demonstrate, using different analytical methodologies, the formation of a BIT–cyclodextrin inclusion complex. Considering that the advantages of microencapsulation involve the potential improvement of the chemical and biological properties of bioactive molecules, a preliminary evaluation of the BIT–cyclodextrin inclusion complex’s antimicrobial and ecotoxicity activity was carried out. Overall, this work provides an innovative solution to improve the efficiency, sustainability, and safety of isothiazolinone-containing biocide formulations.

## 2. Materials and Methods

### 2.1. Chemicals

1,2-Benziothiazol-3(2H)-one (BIT) was supplied by TCI chemicals (Zwijndrecht, Belgium). Cyclodextrins were purchased from Merck Life Science (Algés, Portugal). Deuterated solvents were obtained from Deutero GmbH (Kastellaun, Germany). Additional information about chemicals is available in the Supplementary Materials.

### 2.2. Phase Solubility Studies

Phase solubility studies were performed according to the method described by Higuchi and Connors [19]. Experimental details relating to these studies were included in the Supplementary Materials.

The apparent stability constants, *K*_S_, were calculated, assuming a 1:1 stoichiometry, using Equation (1), in which S_0_ represents the intrinsic solubility of BIT [20]:

*K*_S_ = slope/S_0_ (1 − slope). (1)

Each experiment was performed in triplicate, with the mean (±SD) being reported.

### 2.3. Preparation of Physical Binary Mixture and Inclusion Complex

The physical mixture was prepared by grinding equimolar amounts of BIT and CDs in a mortar until a homogeneous mixture is obtained. The mixture was kept in a closed container protected from light.

BIT inclusion complex was prepared via the kneading method using equimolar amounts of BIT and CDs. Experimental details were included in Supplementary Materials. The dried complex was kept in a closed container, protected from light.

### 2.4. Characterization of the Inclusion Complex

The characterization of the inclusion complex was performed using a combination of experimental techniques.

The UV absorption spectra were obtained using a Shimadzu UV–Vis Spectrophotometer UV-1700 (Kyoto, Japan) in the wavelength range of 200–350 nm. Fourier transform infrared spectroscopy (FTIR) measurements were performed using a Thermo Scientific Nicolet 6700 FTIR (Waltham, MA, USA) spectrometer. Thermogravimetric analysis (TGA) experiments were carried out using a thermogravimetric analyzer (Jupiter STA 449 F3, Netzsch, Selb, Germany). One-dimensional (1D) ^1^H NMR and bidimensional (2D) ROESY spectra were recorded at room temperature at the same final concentration (50 mM) on a Bruker Avance III HD spectrometer (Bruker Scientific LLC, Billerica, MA, USA) operating at 600 MHz. Samples were prepared in 5 vol% DMSO-*d6*/D_2_O. The ^1^H NMR experiments were carried out using a water suppression peak pulse sequence, and chemical shift variations (Δδ) were calculated according to the formula Δδ = δ(complex) − δ(free).

Experimental details relating to these studies were included in Supplementary Materials.

### 2.5. Antibacterial Assays

The antibacterial activity of BIT and inclusion complex on a Gram-negative *Escherichia coli* NTCT 9001 (Selectrol, Buckingham, UK) and a Gram-positive *Staphylococcus aureus* ATCC25923 (Microbiologics, St Cloud, MN, USA) was evaluated using EUCAST macro dilution method, with slight modifications [21,22].

Active cultures of each bacterium were grown in 10 mL Mueller–Hinton II broth (MHB) (Merck Life Science) at 37 ± 0.1 °C overnight. The initial inoculum concentration was set to the equivalent of 0.5 McFarland standard (0.09–0.110 at λ = 600 nm) by dilution in fresh MHB. Samples of BIT, Me-β-CD, and inclusion complex (ranging from 1060 to 118 mg BIT/L) were sterilized by passing through a 0.2 µm syringe filter. The test was performed by adding 100 µL of the inoculum and 900 µL of each sample to 9.0 mL of MHB. Tubes were incubated at 37 ± 0.1 °C for 24 h; afterward, the turbidity was measured at 600 nm.

Positive controls were made by replacing the antibacterial agent with sterile water, and negative controls were made by adding the same volume of lactic acid at 40% (*v*:*v*). The minimum inhibitory concentration (MIC) was determined as the lowest concentration tested that does not present turbidity. The experiments were performed in triplicate.

### 2.6. Ecotoxicity Assay

A BioTox™ WaterTox™ EVO kit (ebpi, Burlington, ON, Canada) was used to carry out the *Aliivibrio fischeri* bioassay. The analytical procedure was performed according to the manufacturer’s instructions and the literature [23,24]. Briefly, the lyophilized bacteria were rehydrated and stabilized first at 4 °C for 30 min and then at 15 °C for another 30 min. A sample dilution series was prepared according to ISO 11348-3:2007 [25], and then 100 μL of each test solution was transferred into a 96-well white plate (Brand, Darmstadt, Germany) in duplicate. *Aliivibrio fischeri* reagent (100 μL) was added immediately before loading of the plate into the luminometer. Luminescence intensity was recorded after 30 s, 15 min, and 30 min using a BioTek Synergy HT multimode reader (Agilent, Santa Clara, CA, USA). The inhibitory effect of the tested chemicals on bacterial luminescence (INH%) was determined according to the literature [23,25]. The EC_50_ values (the chemical concentration which reduces the luminescence of bacteria by 50%) were determined from concentration versus INH% curves [24]. Two reference substances—3.4 mg L^−1^ 3,5-dichlorophenol and 2.2 mg L^−1^ Zn (II) solutions—were also tested in parallel to validate bacterial reagents [23,25].

### 2.7. Statistical Analysis

Statistical analysis of the data was performed using Excel Microsoft 365 software (Microsoft Corp., Redmond, WA, USA). Graphs were plotted using Prism 9, Version 9.5.1 (GraphPad Software, LLC San Diego, CA, USA). Inhibitions and dose–response data (EC_50_ calculations) were evaluated using Microsoft Excel. Data are presented as means ± SD.

## 3. Results and Discussion

### 3.1. Phase Solubility Studies

The most common naturally occurring cyclodextrins are α-cyclodextrin, β-cyclodextrin, and γ-cyclodextrin, comprising six, seven, and eight glucopyranose units, respectively. Due to its greater complexation ability, easy availability, and low-cost, β-CD is the most frequent choice for preparing inclusion complexes. However, since β-CD exhibits relatively low solubility in water, chemically modified derivatives have been developed including hydroxypropyl- and randomly methylated β-cyclodextrin.

The formation of inclusion complexes between BIT and CDs (β-CD, HP-β-CD, and Me-β-CD) was confirmed via UV spectrophotometry. The comparison of the absorption spectra of BIT, in the absence and presence of CDs, showed that the absorbance intensity and consequently the concentration of the compound increased proportionally to the increment of cyclodextrin concentration.

Phase solubility studies of BIT with the different CDs are presented in Figure S1 (see Supplementary Materials). For all CDs, A_L_-type solubility diagrams were obtained, according to the classification established by Higuchi and Connors [19]. The graphs obtained show that the solubility of BIT increases considerably in the presence of cyclodextrins (Figure S1). The slope values were less than one, suggesting the formation of a 1:1 stoichiometric complex in solution.

The stability constants of the inclusion complexes were determined from the plots according to Equation (1) (see Section 2). The stability constants, *K*_S_, calculated for the inclusion complexes of BIT with β-CD, HP-β-CD and Me-β-CD were, respectively, 199.0 ± 2.1, 188.4 ± 3.6, and 299.5 ± 2.9 M^−1^. The intrinsic solubility obtained for BIT from the phase solubility diagrams is quite similar to the data found in the literature for its solubility in water (1.1 g/L at 20 °C, [26]). Table 1 summarizes the data acquired from the phase solubility diagrams: intrinsic solubility (S_0_), slope (α), and stability constant (*K*_S_). From the results, it is possible to conclude that the complexation of BIT with all CDs under study lead to a significant enhancement in its aqueous solubility (see Table 1).

Furthermore, the data suggested that the substitution of the hydroxyl groups of β-CDs, by hydroxypropyl (HP–) and methyl (Me–) functional groups, does not have a considerable influence on the solubility of BIT. These results can easily be explained if we consider that the guest molecule (BIT) is completely encapsulated within the hydrophobic cavity of the β-CDs (Section 3.2.3).

Although the stability constants were similar for all cyclodextrins investigated here, it appears that in aqueous solutions Me-β-CD is a better solubilizer than the other CDs. For this reason, the Me-β-CD was chosen to continue the studies of the inclusion complex in the solid state.

### 3.2. Characterization of the BIT/Me-β-CD Inclusion Complex

#### 3.2.1. Differential Scanning Calorimetry (DSC) Analysis

Thermal methods have been extensively applied in the characterization of cyclodextrin complexes since they can complete the information related to the host–guest molecular inclusion process and the specific properties of the CD complexes. Differential scanning calorimetric (DSC) curves obtained for the samples, BIT, Me-β-CD, physical mixture, and inclusion complex, are shown in Figure S2 (see Supplementary Materials). BIT presents an endothermic peak at its melting temperature (159.1 °C), followed by a peak corresponding to its decomposition nearby 331.5 °C (Figure S2). DSC analysis of Me-β-CD (Figure S2) shows a first broad endotherm peak around 100 °C that corresponds to the water release process [27]. No other significant calorimetric events were observed up to the decomposition of Me-β-CD (363 °C) [27]. In the physical mixture, the peak characteristic of BIT is observed despite appearing at a lower temperature (146.5 °C) and reduced in intensity, which indicates that complexation between BIT and Me-β-CD occurred but is incomplete. On the contrary, the disappearance of the endothermic peak of BIT was observed for the inclusion complex prepared by the kneading method (Figure S2), which may be due to the complete encapsulation of the biocide molecule within the cavity of the Me-β-CD molecule.

#### 3.2.2. Fourier Transform Infrared Spectroscopy (FTIR) Analysis

FTIR is a helpful and widely used tool for studying inclusion complex formation as the variations in the shape, shift, and intensity of the IR absorption peaks of the samples can provide relevant information on their chemical structure [28,29,30]. Figure S3 (see Supplementary Materials) shows the infrared spectra for BIT, Me-β-CD, physical mixture, and inclusion complex of BIT/Me-β-CD. The IR spectrum of Me-β-CD (Figure S3) shows a large absorption band at 3426 cm^−1^ (O–H stretching vibration), 2932 cm^−1^ (C–H stretching vibration), 1638 cm^−1^ (O–H bending vibration) and 1158, 1086, and 1046 cm^–1^ (C–O– stretching compatible to the bonds on ether and hydroxyl groups). The spectrum of BIT (Figure S3) shows characteristic bands at 3457 cm^−1^ (N–H stretching), 1652 cm^−1^ (C=O stretching), 1443 cm^−1^ (N–H bending), 1148 cm^−1^ (C–O stretching), and C–S stretching vibrations in the region 750–600 cm^−1^. In the spectra of the BIT/Me-β-CD physical mixture and inclusion complex, no new peaks were observed. The FTIR spectrum of the physical mixture was essentially a combination of the spectra of Me-β-CD and BIT. In contrast, in the spectrum of the complex, some characteristic absorption bands of BIT were shifted, decreased, or disappeared. This happened for the bands of the carbonyl and amine groups, which suffered a significant decrease, shift, and/or disappearance (Figure S3). These changes can be the result from the vibrational constraints occurred upon the inclusion of BIT within the Me-β-CD cavity [30].

#### 3.2.3. Nuclear Magnetic Resonance (NMR) Spectroscopy Analysis

Nuclear magnetic resonance (NMR) has been widely used to study the structure and dynamics of molecular systems, playing a fundamental role in characterizing host–guest complexes and other supramolecular systems. If an inclusion process occurs, it will affect the proton chemical shifts (δ) of the guest and the host due to a change in the chemical environment [29,31,32,33].

**Figure 2:**
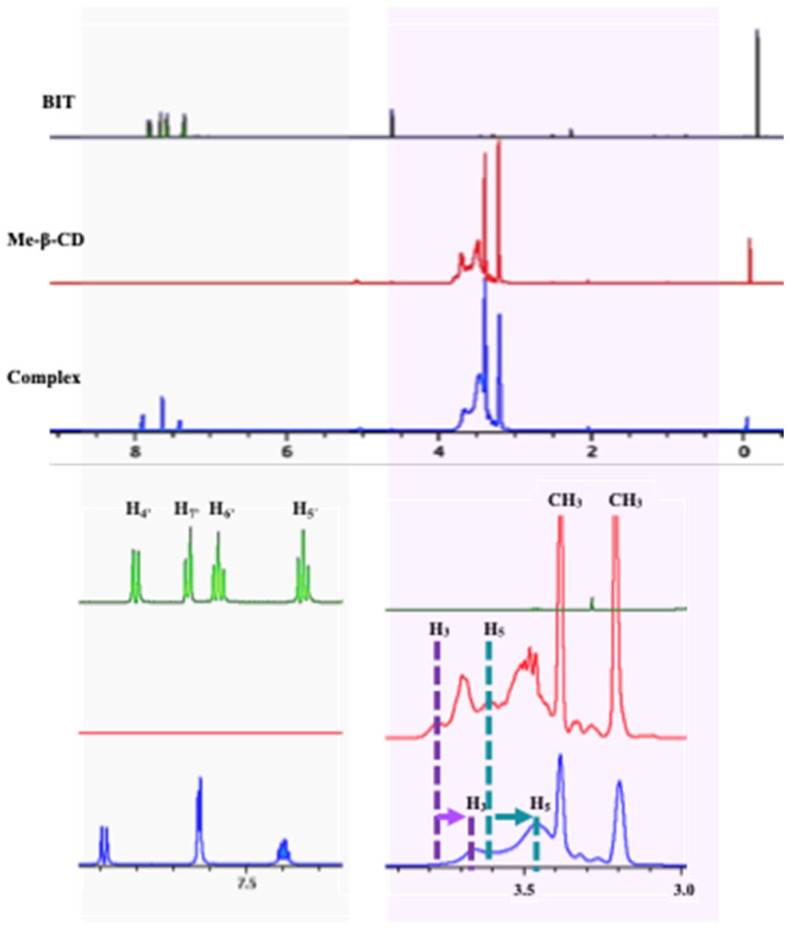
^1^H NMR spectrum of the BIT, Me-β-CD, and BIT/Me-β-CD inclusion complex in DMSO-*d6*/D_2_O.

To confirm the formation of the inclusion complex, a 1D ^1^H NMR study was performed (Figure 2). The chemical shift values observed in the spectra of the Me-β-CD, BIT, and inclusion complex are depicted in Table 2. The assignments of Me-β-CD and BIT were in accordance with the literature [34,35].

^1^H NMR spectrum of the inclusion complex showed significant changes in the chemical shifts of the host and guest protons (Table 2). Upon complexation, the H-3 and H-5 proton signals of the Me-β-CD cavity inner protons showed the most pronounced upfield shifts.

As both protons are inside the CD cavity, the data suggest a clear involvement of these protons in the host−guest interactions. Protons H-1, H-2, H-4, and -CH_3_ of Me-β-CD experienced smaller or negligible chemical shift changes (Table 2). As H-3 and H-5 protons are positioned at different locations of the internal cavity of the guest (H-3 protons are near the wide rim and H-5 protons are close to the narrow side), the observed magnitude of their displacements can provide additional information about the CD inclusion complex. As the absolute value of Δδ H3 ≤ Δδ H5, it is possible to assume a total inclusion of the guest molecule (BIT) inside the cavity of the cyclodextrin [31,33]. The chemical shifts of BIT aromatic protons were also affected, showing significant downfield shifts and alterations in their spin–spin coupling pattern due to the variation of the chemical environment caused by the complex formation (Table 2).

**Figure 3:**
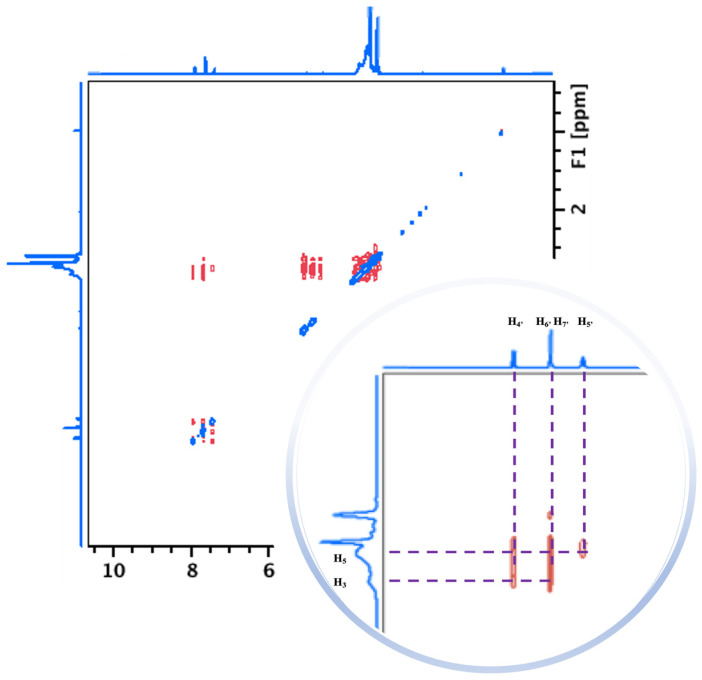
The 2D-ROESY spectrum of BIT/Me-β-CD inclusion complex in DMSO-*d6*/D_2_O and a zoomed view of cross-peak region.

To confirm the 1D-NMR data, a two-dimensional (2D) ROESY (rotating-frame Overhauser enhancement spectroscopy) NMR experiment was conducted (Figure 3). The 2D ROESY provides information regarding the spatial proximity of the protons present in the host and guest molecules, allowing for the confirmation of the complex formation [31]. If the guest has been included inside the cyclodextrin cavity, the protons of the guest and H-3 and H-5 protons of the cyclodextrin come closer than 4 Å, and cross-peaks will be observed in the spectra [36].

The intermolecular cross-peaks observed between BIT aromatic protons (H-4′, H-5′, H-6′, H-7′) and the H-3 and H-5 protons of Me-β-CD confirm the complex formation. Strong interactions between H-6′ and H-7′ of BIT, when compared with those from H-4’ and H-5′ protons, were observed with H-3 and H-5 protons of Me-β-CD, which are consistent with the changes observed in 1D NMR (Figure 3). The analysis also showed that the interaction of BIT H-5′ aromatic proton with H-5 from Me-β-CD is the weaker interaction detected [37].

**Figure 4:**
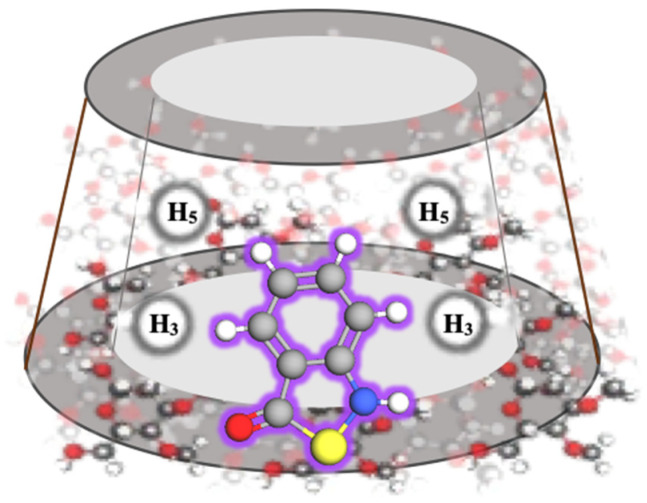
Schematic representation of BIT/Me-β-CD inclusion complex.

Weighing up all the interactions from the ROESY spectrum, it is plausible to deduce the complete insertion of BIT into the hydrophobic cavity of Me-β-CD, with the thiazol-3(2H)-one fragment being oriented towards the wide rim of Me-β-CD (Figure 4).

### 3.3. Antibacterial Activity

Antibacterial assays are important tools with which to test and screen the inhibitory effects of myriad compounds against microorganisms before establishing their inhibitory spectra. A variety of laboratory methods can be used to evaluate or screen the in vitro antimicrobial activity of an extract or a pure compound. One of the most well-known is the broth macrodilution method [21,22,38].

**Figure 5:**
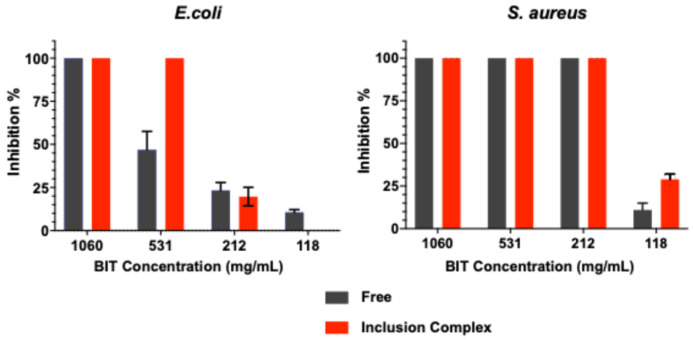
Inhibition growth percentage achieved by free BIT and BIT/Me-β-CD inclusion complex on *E. coli* and *S. aureus* strains (average of three replicates).

The antibacterial activity of BIT and BIT/Me-β-CD inclusion complex was tested against Gram-negative (*E. coli*) and Gram-positive (*S. aureus*) bacteria (Figure 5). The results showed that both tested organisms were susceptible to the compounds and that the inclusion complex displayed a higher antibacterial activity, compared to BIT.

It was observed that *S. aureus* was completely inhibited by both BIT and the BIT/Me-β-CD inclusion complex in the range between 1060 and 212 mg/L. The biocide and the inclusion complex showed similar antibacterial activity for *S. aureus*, with a minimal inhibitory concentration (MIC) of 212 mg/L.

Both BIT and the inclusion complex showed a lower activity against *E. coli* than to *S. aureus*. A MIC value of 1060 mg/L was obtained for BIT, while the inclusion complex displayed a higher antibacterial activity against this strain, with a MIC value of 531 mg/L. Since no antibacterial effect associated with Me-β-CD was observed in this work, the greater antibacterial activity noticed for BIT/Me-β-CD may be attributed to the increased solubility of the inclusion complex, allowing BIT to be more available for membrane transportation and to become more accessible for specific tissues [39]. The difference in the susceptibility of the microorganisms tested can be related to the structure of the cell wall since the outer membrane of Gram-negative bacteria serves as a barrier against a variety of external chemicals, including antibiotics [40]. The cytotoxicity of the inclusion complex may be associated with increased cell permeability and membrane disorganization caused by solubilization of membrane sterols [41]. However, interference of Me-β-CD in bacterial quorum sensing cannot be excluded (Section 3.4).

Minimum bactericidal concentration was not determined since bacterial growing was still observed in Petri dishes, prepared from the non-growing tubes, at the maximum concentration tested (1060 mg/L), which indicates that BIT acts as a bacteriostatic agent.

### 3.4. Ecotoxicity Profile

Manifold conventional methods for testing the ecotoxicological effects on terrestrial and aquatic environments are available and include a broad range of model organisms. The luminescent bacteria test using *Aliivibrio fischeri* is a widely accepted bacterial toxicity test and has been normalized by the International Organization for Standardization (ISO) [25]. This bioassay has been widely applied for toxicity monitoring due to multiple advantages that include shorter test duration, sensitivity, cost-effectiveness, and ease of operation, and also because it is applicable for a huge variety of environmental and industrial samples [42]. The luminescent bacteria test detects the inhibitory effect of chemical substances on the emission intensity of *Aliivibrio fischeri*. In opposition, a decrease in emission intensity indicates metabolic disruption of the bacteria [25].

The reliability of the experimental procedure was evaluated by estimating the toxicity of two reference compounds, 3,5-dichlorophenol and Zn (II) solution. The results obtained were comparable with data published in the literature [24].

**Figure 6:**
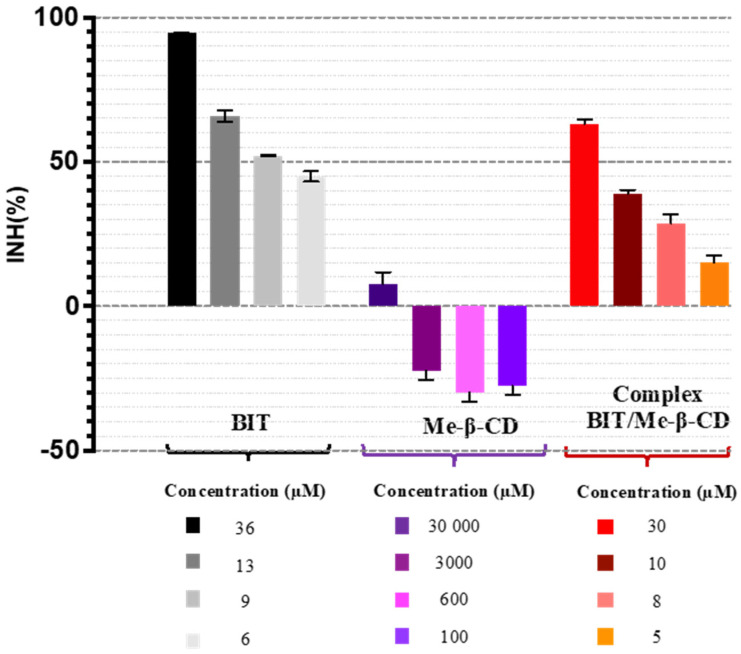
Effect of increasing concentrations of BIT, Me-β-CD, and BIT/Me-β-CD inclusion complex on the inhibition of *A. fischeri* bioluminescence (average of three replicates).

The toxicity of the biocide was estimated using different incubation times. The results showed that BIT was already toxic to *A. fischeri* during the first seconds of exposure, indicating that this biocide is a fast-acting toxicant to bacteria. No significant difference in the 30 s and 15 min EC_50_ values was observed. The data found are consistent with the behavior described in the literature for other low-molecular-weight organic toxicants [24,43]. The EC_50_ value obtained for BIT was 1.08 mg/L (Figure 6). The value obtained is comparable to that described in the literature for another commercial isothiazolinone biocide: 2-methyl-4-isothiazolin-3-one (MIT; EC_50_ = 1.61 mg/L) [4].

The effect of Me-β-CD on the bioluminescence of *A. fischeri* was assessed in a series of experiments in 0.1–30 mM concentration range. The Me-β-CD exhibited stimulatory effects (<30%) on bioluminescence for the lower concentrations at all exposure times tested (Figure 6). A slight inhibitory effect is seen at the higher concentration tested (30 mM). The data obtained are consistent with findings reported in the literature [41]. The quorum quenching effect of cyclodextrins in the *A. fischeri* bioluminescence intensity was recently demonstrated [44]. It has been shown that β-CD and its derivatives decreased the bioluminescence from a slight to a medium level and that the inhibition level increased with time and with growing cyclodextrin concentrations. This CD-modulated quorum quenching is a novel approach, and the available information on this topic refers mainly to their effect on processes regulated by bacterial communication. Cyclodextrins can influence bacterial communication by forming inclusion complexes with signaling molecules involved in bacterial communication, with the extent of this effect depending on the cyclodextrin’s cavity size and substituents [45].

The effect of the BIT/Me-β-CD inclusion complex on the bioluminescence of *A. fischeri* was tested, and although this study has shown a clear dose–effect correlation, the data found were considered to be inconclusive due to the biostimulation and quorum quenching effect of the cyclodextrin.

## 4. Conclusions

The inclusion complexation behavior, characterization, and binding ability of BIT with different β-CDs (β-CD, HP-β-CD, and Me-β-CD) was investigated. The data obtained showed that this biocide can form inclusion complexes with the cyclodextrins both in an aqueous solution and in a solid state. Phase solubility profiles indicate that the solubility of BIT significantly increased in the presence of β-CDs. The stability constants obtained for the inclusion complexes of BIT with the studied β-CDs ranged between 188.4 and 299.5 M^−1^ for HP-β-CD and Me-β-CD, respectively.

The increase in the BIT solubility was attributed to the inclusion complex formation with β-CDs, as demonstrated by UV–Vis, FTIR, DSC, and NMR (^1^H and 2D ROESY) methods. The results obtained by NMR confirm BIT entrapment into the cavity of the CDs.

Furthermore, in vitro studies demonstrated an enhancement of the antibacterial profile of the inclusion complex, thereby increasing the possibility of using the inclusion compound in commercial products in order to improve BIT biocide effect. From an ecological point of view, microencapsulation seems to diminish the harmful effects of BIT; however, due to the quorum quenching effect exhibited by β-CDs, further investigation is needed.

## Figures and Tables

**Table 1:** Intrinsic solubility of BIT (S_0_), slope (α), and stability constant (*K*_S_).

	S_0_ ± SD (10^−3^ M)	α ± SD	*K*_S_ ± SD (M^−1^)	R^2^
β-CD	7.60 ± 0.12	0.602 ± 0.006	199.0 ± 2.1	0.9940
HP-β-CD	7.96 ± 0.30	0.600 ± 0.014	188.4 ± 3.6	0.9980
Me-β-CD	7.94 ± 0.28	0.704 ± 0.008	299.5 ± 2.9	0.9990

**Table 2:** ^1^H NMR chemical shifts of Me-β-CD, BIT, and inclusion complex.

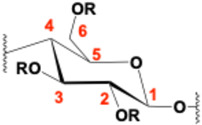				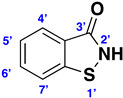			
**Proton**	**δ Me-β-CD**	**δ Complex**	**Δ** δ	**Proton**	**δ BIT**	**δ Complex**	**Δ** δ
**H-1**	5.0718	5.0331	−0.04	**H-4′**	7.8014 (*d*, *J* = 8.2)	7.8877 (*d*, *J* = 8.2)	+0.09
**H-2**	3.3360	3.3180	−0.02	**H-5′**	7.3428 (*dd*, *J* = 8.2; 8.0)	7.3963 (*m*)	+0.05
**H-3**	3.7720	3.6550	−0.12	**H-6′**	7.5755 (*ddd*, *J* = 8.0; 8.0; 1.2)	7.6278 **	+0.05
**H-4**	3.2870	3.26750	−0.02	**H-7′**	7.6584 (*d*, *J* = 8.0)	7.6278 **	−0.03
**H-5**	3.6080	3.4542	−0.15				
**H-6**	3.6970	n.a *	-				
**OCH_3_**	3.3811	3.3843	+0.003				
**OCH_3_**	3.2086	3.1966	−0.012				

n.a * = not assigned due to the overlapping of signals. ** The chemical shift of H-6′ and H-7′ of the complex is the mean of both signals due to their overlapping.

## Data Availability

The data presented in this study are available on request from the corresponding author.

## Supplementary Materials

The following supporting information can be downloaded at https://www.mdpi.com/article/10.3390/toxics12090674/s1: The supporting information includes experimental details, the phase solubility diagrams, DSC thermograms and FTIR spectra. Figure S1: Phase solubility diagrams of BIT with the different cyclodextrins, in aqueous solution at 25 ± 2°C.; Figure S2: DSC thermograms of BIT/Me-β-CD system; Figure S3: FTIR spectra of BIT/Me-β-CD system.
