# Supplementary material for: Microencapsulation of the Biocide Benzisothiazolinone (BIT) by Inclusion in Methyl-β-cyclodextrin and Screening of Its Antibacterial and Ecotoxicity Properties
Source: Toxics. 2024 Sep 16;12(9):674. doi: 10.3390/toxics12090674 (PMC11435904; doi:10.3390/toxics12090674)
Supplement: Supplementary file 1 [file toxics-12-00674-s001.zip › toxics-3081119-supplementary.pdf]

Microencapsulation of the Biocide Benzisothiazolinone (BIT) by Inclusion in Methyl–β–cyclodextrin and Screening of Its Antibacterial and Ecotoxicity Properties

Vânia F.M. Silva[a], Aurora Silva[b,c], E. Manuela P.J. Garrido[a,d], Fernanda Borges[e], Alexandra Gaspar[e*], and Jorge M.P.J. Garrido[a,d*]

[a] CIETI, ISEP, Polytechnic of Porto, 4249-015 Porto, Portugal.

[b] Nutrition and Bromatology Group, Department of Analytical and Food Chemistry, Faculty of Food Science and Technology, University of Vigo, Ourense Campus, E32004 Ourense, Spain.

[c] REQUIMTE/LAQV, ISEP, Polytechnic of Porto, 4249-015 Porto, Portugal.

[d] CIQUP-IMS, ISEP, Polytechnic of Porto, 4249-015 Porto, Portugal.

[e] CIQUP-IMS, Department of Chemistry and Biochemistry, Faculty of Sciences, University of Porto, 4169–007 Porto, Portugal.

Table of Contents of Supporting Information:

S1. Supplementary Materials and Methods

S.1.1 Chemicals

S.1.2 Phase Solubility Studies

S.1.3 Preparation of Physical Binary Mixture and Inclusion Complex

S.1.4 Characterization of the Inclusion Complex

S2. Supplementary Discussion

S2.1. Phase Solubility Diagrams of BIT

S2.2. DSC Thermograms of BIT/Me-β-CD System

S2.3. FTIR Spectra of BIT/Me-β-CD System

S1. Supplementary Materials and Methods

S.1.1 Chemicals

1,2-Benziothiazol-3(2H)-one (BIT) was supplied by TCI chemicals (CAS No. 2634-33-5, C_7H_5NOS, MW 151.18, melting point 155.0 to 160.0°C, purity > 98.0% (GC)). β-Cyclodextrin (β-CD; ≥98.0% (HPLC); M_w 1134.98), (2-hydroxypropyl)-β-cyclodextrin (HP-β-CD; 0.8 molar substitution; average M_w ~1460), and methyl-β-cyclodextrin (Me-β-CD; 1.6-2.0 mol CH_3 per unit anhydroglucose; average M_n ~1310) were purchased from Merck Life Science (Algés, Portugal). All other reagents and solvents were pro analysis grade and used without additional purification. Deionized water (conductivity < 0.1 $\mu S\ cm^{-1}$) was used throughout all the experiments. Stock solutions of the tested chemicals (BIT, Me-β-CD, and inclusion complex) were prepared in 1% DMSO (for antibacterial assays) and MilliQ water (for ecotoxicity assay) and stored in the dark. Before use, the stock solutions were diluted in 1% DMSO (for antibacterial assays) or 2% NaCl (for ecotoxicity assay).

S.1.2 Phase Solubility Studies

An excess amount of BIT (150 mg) was added to 25 mL of aqueous solutions containing increasing concentrations of β-CD (0 – 8 mM), HP-β-CD, and Me-β-CD (0 – 30 mM). Then, the suspensions were shaken on a rotary shaker (Ika KS 4000i, Germany) at 25 ± 2 °C for 48 hours. After equilibrium was reached, suspensions were filtered through a 0.45 µm membrane filter (Millipore) and properly diluted. The concentration of BIT was determined by spectrophotometry (Shimadzu UV–Vis Spectrophotometer, UV-1700, Japan) at 226 nm. The UV absorption of all cyclodextrins was negligible at the assay wavelength.

S.1.3 Preparation of Physical Binary Mixture and Inclusion Complex

The BIT inclusion complex was prepared using the kneading method, as described elsewhere [31]. Briefly, equimolar amounts of BIT (1 mM) and CDs were accurately weighed, transferred to a mortar, and kneaded for 30 minutes. During this process, a small volume of ethanol (ca.1 mL) was added to the mixture to maintain a suitable consistency. The final mixture was dried overnight, at room temperature, in a desiccator under vacuum to remove traces of solvent.

S.1.4 Characterization of the Inclusion Complex

Fourier transform infrared spectroscopy (FTIR) measurements were performed using a Thermo Scientific Nicolet 6700 FTIR (Waltham, MA, USA) spectrometer in the 4,000–400 cm^{-1} spectral range with a resolution of 1 cm-1 using the KBr pellet method.

The thermal behavior was studied by heating the samples in a sealed aluminum sample pan from 25 °C to 400 °C at a rate of 10 °C/min and under a nitrogen flow of 70 mL/min.

One-dimensional (1D) 1H NMR and bidimensional (2D) ROESY spectra were recorded at room temperature on a Bruker Avance III HD spectrometer operating at 600 MHz. The chemical shifts were expressed in δ (ppm) values and were relative to DMSO-d6 (δ 2.5 ppm). Coupling constants (J) were given in Hz.

Bidimensional ROESY spectra were obtained under the following conditions: 40 scans, a mixing time of 350 ms, a spectral width of 12.02 ppm, and a relaxation delay of 2s.

S2. Supplementary Discussion

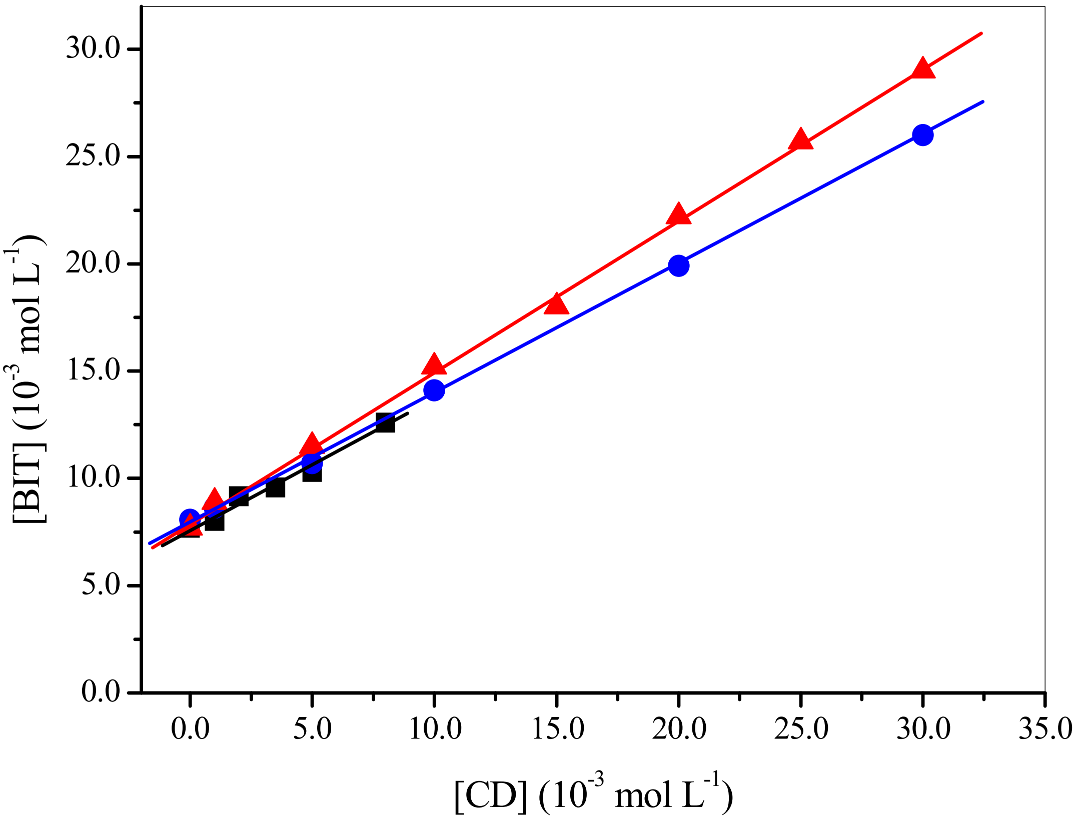

Figure S1. Phase solubility diagram of BIT with (■), ▬ β-CD, (●), ▬ HP-β-CD and (▲), ▬ Me-β-CD in aqueous solution at 25 ± 2°C. Values are mean ± SD (n = 3).

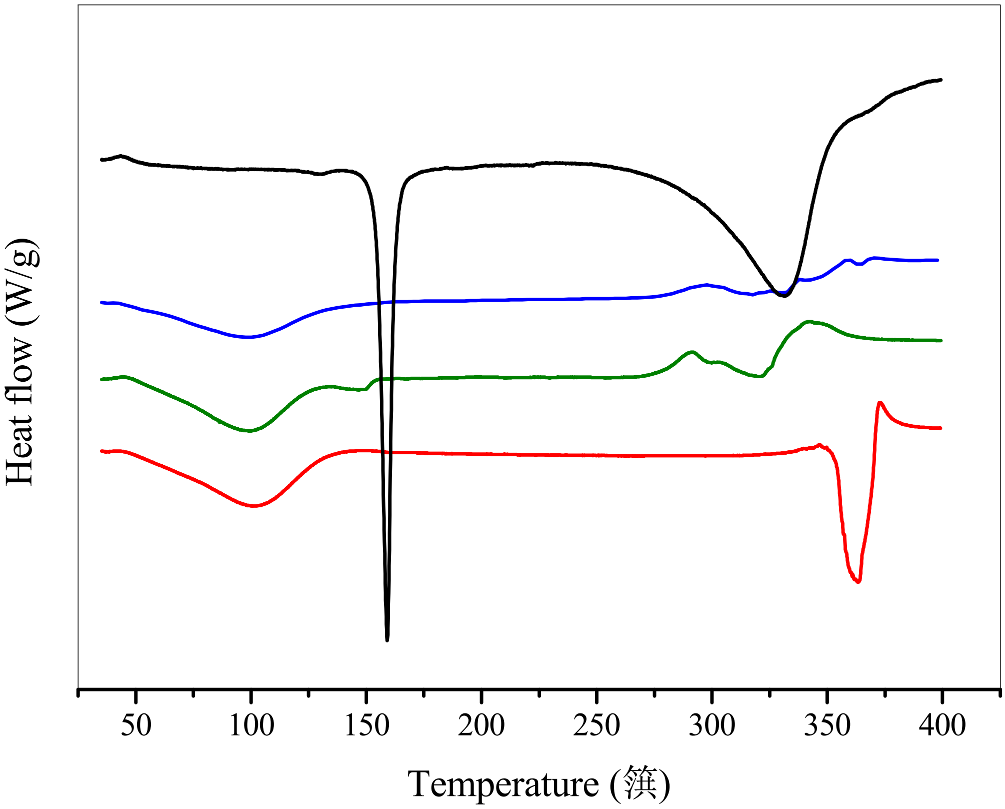

Figure S2. DSC thermogram of BIT/Me-β-CD system: (▬) BIT, (▬) Me-β-CD, (▬) physical mixture, and (▬) inclusion complex.

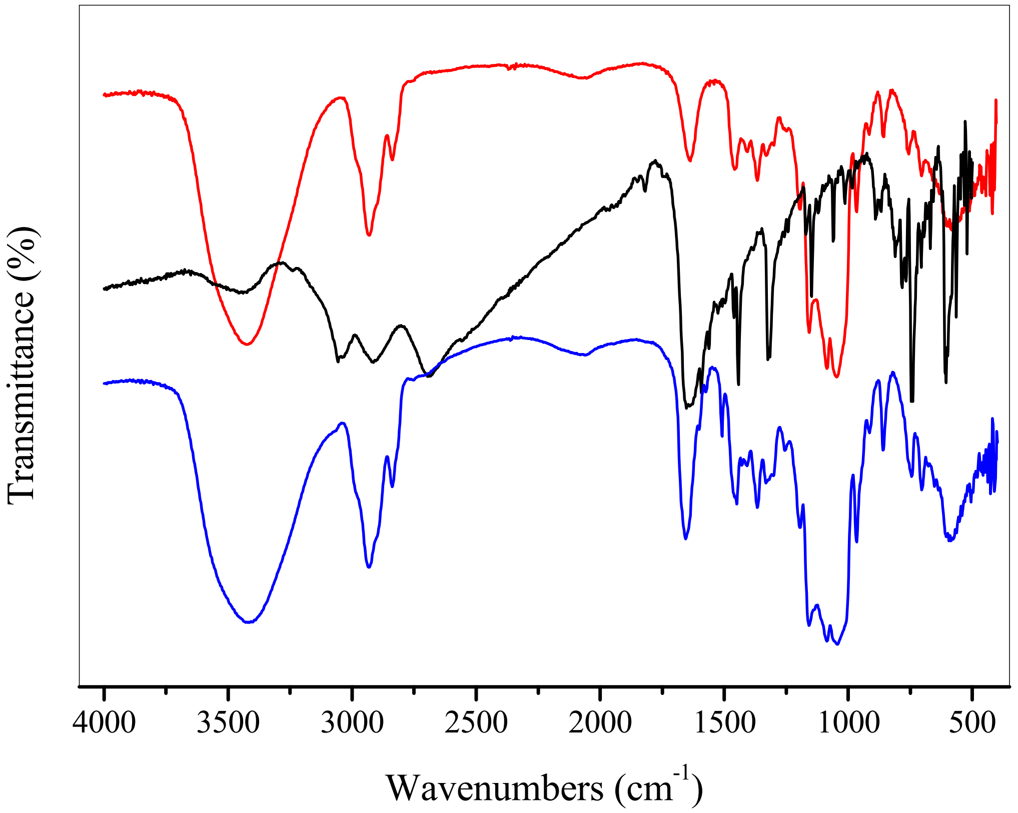

Figure S3. FTIR spectra of BIT/Me-β-CD system: (▬) BIT, (▬) Me-β-CD, and (▬) inclusion complex.
